# Synthesis and characteristics of NH_2_-functionalized polymer films to align and immobilize DNA molecules

**DOI:** 10.1186/1556-276X-7-30

**Published:** 2012-01-05

**Authors:** Hyung Jin Kim, In-Seob Bae, Sang-Jin Cho, Jin-Hyo Boo, Byung-Cheo Lee, Jinhee Heo, Ilsub Chung, Byungyou Hong

**Affiliations:** 1 School of Information and Communication Engineering, Sungkyunkwan University, Suwon, 440-746, Republic of Korea; 2 Metal Development Group, MDS Development Team, MDS Division, Samsung Techwin Co., LTD 42, Changwon, 642-716, Republic of Korea; 3 Department of Chemistry and Institute of Basic Science, Sungkyunkwan University, Suwon, 440-746, Republic of Korea; 4 Quantum Optics Lab, Korea Atomic Energy Research Institute, Daejeon, 305-353, Republic of Korea; 5 Korea Institute of Materials Science, Changwon, 641-831, Republic of Korea

**Keywords:** DNA molecules, NH_2_-functionalized polymer thin films, aniline-capped gold nanoparticles, gold nanowires.

## Abstract

We developed a method to use NH_2_-functionalized polymer films to align and immobilize DNA molecules on a Si substrate. The plasma-polymerized cyclohexane film was deposited on the Si substrate according to the radio frequency plasma-enhanced chemical vapor deposition method using a single molecular precursor, and it was then treated by the dielectric barrier discharge method in a nitrogen environment under atmospheric pressure. Changes in the chemistry of the surface functional groups were studied using X-ray photoelectron spectroscopy and Fourier transformed infrared spectroscopy. The wettability of the surfaces was examined using dynamic contact angle measurements, and the surface morphology was evaluated using atomic force microscopy.

We utilized a tilting method to align λ-DNA molecules that were immobilized by the electrostatic interaction between the amine groups in NH_2_-functionalized polymer films and the phosphate groups in the DNA. The DNA was treated with positively charged gold nanoparticles to make a conductive nanowire that uses the DNA as a template. We observed that the NH_2_-functionalized polymer film was useful for aligning and immobilizing the DNA, and thus the DNA-templated nanowires.

## Introduction

In the field of nanotechnology, DNA molecules are considered attractive building blocks for generating superstructures because the DNA itself is a nanowire with a nanoscale diameter of approximately 2 nm, and it has a very long linear structure with a well-defined polymeric sequence and many functional groups [1,2]. Therefore, the metal nanowires that are fabricated by the conjugation of DNA molecules and nanosize metal nanoparticles have been extensively investigated for their application to the highly ordered electronic components of nanocircuitry and/or nanodevices [3-7]. The technique of aligning and immobilizing the DNA molecules on various substrates is an important technique; however, this technique is very difficult to control. The technique of controlling and manipulating DNA molecules with nanometer resolution is a subject of priority in the field of DNA-based nanotechnology; many research groups have studied the application of the latter technique in nanotechnology [8]. Recently, it became possible to align and immobilize DNAs by various physical methods such as electric or dielectric force [9], microcontact printing (μCP) [10,11], fluid flow [12], and molecular combing/surface tension [13,14]. Surface modification, such as silanization, has also been used to assist the immobilization and the alignment of DNA molecules [14-17]. However, self-assembled monolayers that are formed by the silanization method have unstable physical and chemical conditions because they depend on conditions such as temperature and humidity during coating.

In this paper, we present a new procedure to reproducibly align and immobilize DNAs using NH_2_-functionalized polymer films that are deposited on a Si substrate according to the radio frequency [RF] plasma-enhanced chemical vapor deposition [PECVD] method using cyclohexane, and it is then treated by the dielectric barrier discharge [DBD] method in a nitrogen [N_2_] environment. The key point of this technique is the alignment of the DNA through the interactions between the phosphate groups in the DNA and the amine groups on the surface of the NH_2_-functionalized polymer film. It is observed on how well the DNA is arranged on the polymer film's surface by atomic force microscopy [AFM] examination; moreover, we discuss the feasibility of this technique for nanowire synthesis for its application to nanodevices.

## Experimental details

A solution of DNA (16 μm long - 48,502 bp; Bio Basic Inc., Markham, Ontario, Canada) was prepared in a concentration of 10 ng/μL with TE buffer (10 mM tris-HCl and 1 mM EDTA, pH 8.0). Aniline (Sigma-Aldrich Corporation, St. Louis, MO, USA)-capped Au nanoparticles [AN-AuNPs] were prepared based on the conventional reduction of HAuCl_4 _(Sigma-Aldrich Corporation, St. Louis, MO, USA) using aniline as a reducer [14,17,18]. The plasma-polymerized cyclohexane films, with an average thickness of 200 nm, were deposited on the Si substrate at a RF power of 20 to approximately 50 W according to the PECVD method using cyclohexane, and these films were then treated by the DBD method in a N_2 _environment under atmospheric pressure at a RF power of 150 W for 60 s to form the amine groups on the polymer film surface. The NH_2_-functionalized polymer surface plays an important role in attaching the DNA onto the substrate through a strong electrostatic interaction between the amine groups of the sample surface and the negatively charged phosphate backbone of the DNA [18]. Changes in the chemistry of the surface functional groups were studied using X-ray photoelectron spectroscopy [XPS] and Fourier transform infrared spectroscopy [FT-IR] spectroscopy. The wettability of the surfaces was examined using dynamic contact angle measurements, and the surface morphology was evaluated using AFM (SPA 400, SII Nanotechnology Inc., Sunto-gun, Shizuoka, Japan).

DNA alignment was carried out according to the tilting method, and this resulted in highly aligned DNA patterns on the substrate [16,18]. A λ-DNA solution of 10 μL was dropped on the surface-treated polymer film, and the sample was then tilted to almost 90°. DNA molecules were stretched and aligned along the direction of the sample's tilting. For the synthesis of the conductive nanowire, DNA molecules were treated with AN-AuNPs of 60 μL for 30 min; AN-AuNPs of 60 μL were prepared based on the conventional reduction of HAuCl_4 _using aniline as a reducer [14,17,19]. Next, the sample is rinsed with double distilled water. During this treatment, the DNA-templated gold nanowires [AuNWs] are fabricated through the electrostatic interaction between the positively charged amine groups of the AuNPs and the negatively charged phosphate groups in the DNA. The observation by AFM showed that the DNAs stretched on the NH_2_-functionalized polymer films and that the AuNPs were assembled along DNA molecules.

## Results and discussions

The purpose of this work was to confirm the possibility of aligning and immobilizing DNA molecules on the NH_2_-functionalized polymer thin films formed by the conventional PECVD and DBD methods; this was motivated by the possibility that the negatively charged phosphate backbone of the DNA would electostatically interact with the positively charged polymer film with an amine group.

**Figure 1:**
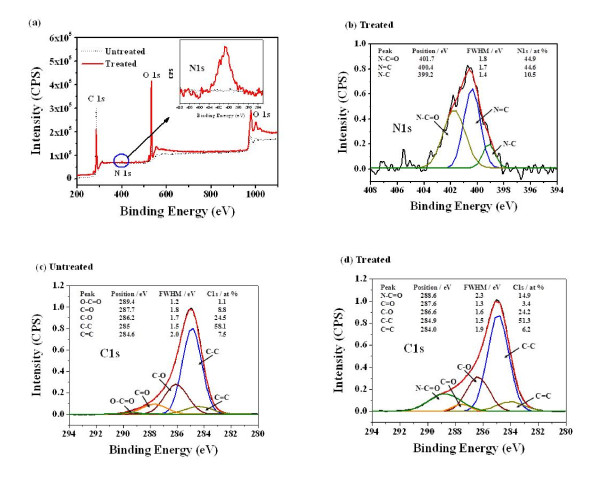
**XPS spectra**. (**a**) A wide scan XPS spectra of the N_2 _the plasma-untreated and treated polymer films. The inset shows enlarged N1s XPS spectra in Figure 1. (**b**) High-resolution XPS spectra of N1s peaks for the plasma-treated polymer film. (**c**) High-resolution XPS spectra of C1s peaks for the plasma-untreated polymer film. (**d**) High-resolution XPS spectra of C1s peaks for the N_2 _plasma-treated polymer film.

XPS spectra in wide scan and high resolution were recorded for the plasma-untreated and plasma-treated polymer thin films, which were deposited on the Si substrate at a RF power of 30 W by the PECVD method using cyclohexane, and then, they were treated by DBD method in N_2 _at a RF power of 150 W for 60 s to form the functional groups on the surface region and to show the subsequent changes of the chemistry on the surface that were introduced by the N_2 _plasma treatment. Figure 1a mainly shows the signals in XPS that correspond to O1s (532 eV), C1s (285 eV), and N1s (400 eV). The inset in Figure 1a shows enlarged N1s XPS spectra for the plasma-treated and untreated polymer films. As seen in Figure 1a, there were no N1s signals on the plasma-untreated polymer films. However, N_2 _plasma-treated polymer films showed N1s signals and strong reduction of the C1s signal. The N1s peak for the N_2 _plasma-treated polymer film was resolved into three peaks at 399.2, 400.4, and 401.7 eV (Figure 1b). These peaks correspond to N-C, N = C, and N-C = O with relative compositions of 10.5%, 44.6%, and 44.9%, respectively [18,20]. This indicates the presence of nitrogen atoms with at least three different binding energies.

Details of different carbon functionalities at the surface were also determined from the C1s high-resolution XPS spectrum in Figure 1c, d. The C1s spectra for the untreated and treated polymer films were deconvoluted into five component peaks, which were C = C at 284.6 eV, C-C at 285 eV, C-O at 286.2 eV (includes a small C-N bond contribution at 285.9 eV), C = O at 287.7 eV (includes a small C = N bond contribution at 288.0 eV), and O-C = O at 289.4 eV [18-21].

Due to the N_2 _plasma treatment, there was a significant incorporation of nitrogen-containing groups. The O1s signal was unaffected; however, it was likely that the incorporation of oxygen moieties occurred upon exposure to air before the plasma treatment, but at the expense of oxygenated molecules at the surface lost during plasma exposure. Consequently, the intensity of the C1s spectrum was affected such that the intensities of C = O and O-C = O bonds decreased. This was a concomitant increase of the peak intensity at 288.6 eV corresponding to the O = C-N bond. It indicates significant incorporation of nitrogen-containing groups, but the exact identification of functionalities that are bound to the surface is difficult. In addition to C-O type moieties, the component at 286.6 eV may include C-NH_2_, C-NH, or C-N = C groups. The N-C = O peak located at 288.6 eV could imply N-C-O and CONH_2 _type functionalities. The results were likely that nitrogen-containing species have been formed in the N_2 _plasma-treated polymer film (Figure 1).

**Figure 2:**
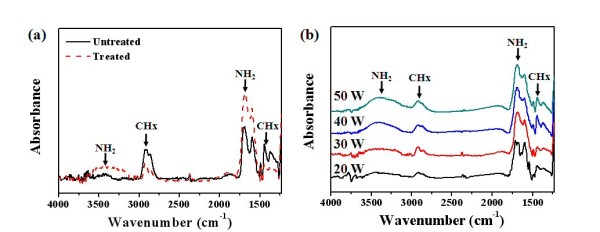
**FT-IR spectra**. (**a**) FT-IR spectra of the N_2 _plasma-untreated and treated polymer films at a RF power of 150 W for 60 s. (**b**) FT-IR spectra for N_2 _plasma-treated polymer films that were synthesized with the varied RF power (20 to approximately 50 W).

Figure 2 shows the attenuated total reflection method of the FT-IR absorption spectra over the range of 600 to approximately 4,000 cm^-1 ^on the plasma-untreated and plasma-treated polymer films according to the DBD plasma treatment method. The spectra exhibit an absorption peak for increasing the N-H stretching vibration at 3,400 cm^-1 ^and the N-H bending vibration at 1,650 cm^-1^, and the spectra exhibit an absorption peak for decreasing the C-H stretching vibration at 2,900 cm^-1 ^and the C-H bending vibration at 1,400 cm^-1 ^in the case of the N_2 _plasma-treated polymer film, as shown in Figure 2a. The results further prove that the nitrogen atoms were covalently grafted to the polymer main chains to form new amine groups (NH_2_), which correspond to the XPS results, because N ions are chemically grafted onto the polymeric structure and are highly reactive with H ions.

**Figure 3:**
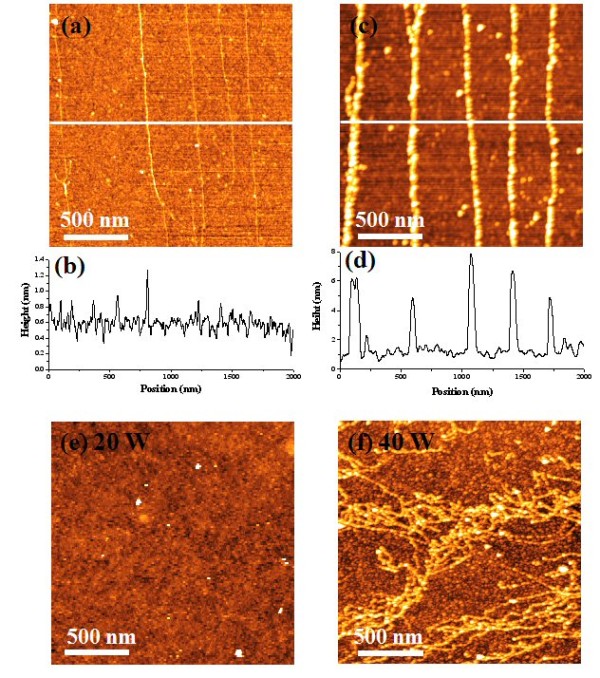
**AFM images**. (**a**) AFM images of the DNA molecules that were stretched and immobilized on the N_2 _plasma-treated polymer film synthesized with 30 W of RF power. (**b**) The scan profile along the white line of the image (a). (**c**) AFM image of AuNWs formed along the stretched DNA molecules on the N_2 _plasma-treated polymer film that was synthesized with 30 W of RF power. (**d**) The scan profile along the white line of image (c). (**e**, **f**) AFM images showing the dependence of DNA stretch and alignment on NH_2_-functionalized polymer films that were synthesized (e) at 20 W power and (f) at 40 W power, respectively, and then treated with DBD at a RF power of 150 W for 60 s.

The average surface roughness of the NH_2_-functionalized polymer films, which were deposited on the Si substrate at a RF power of 30 W by PECVD method using cyclohexane and then treated by the DBD method in N_2 _at a RF power of 150 W for 60 s, was 0.45 ± 0.03 nm according to the AFM measurements. This smooth surface simplified the AFM measurements of the well-aligned DNA on the Si wafer. Tilting the sample induced an air-water interface motion, and thus, the DNA molecules were aligned along the motion direction of the droplet. The average height of the aligned DNA molecules was 0.41 ± 0.17 nm (Figure 3b), and this value was consistent with the height of a double-stranded DNA from the AFM measurements that were reported by other groups [10,11]. λ-DNAs were confirmed to be aligned on the NH_2_-functionalized polymer films as expected, and they were, more or less, evenly distributed.

In addition to the alignment and immobilization of DNA molecules on the NH_2_-functionalized polymer film, another notable point proposed in this work is that the conductive AuNWs can be formed by the treatment of DNAs with the aniline [AN]-capped AuNPs. DNAs aligned on the NH_2_-functionalized polymer film were treated by a solution of AuNPs (5 nm) whose surface was positively charged after they were treated with AN solution, and thus, AuNPs were strongly attached to the negatively charged DNAs. However, they were weakly bound on the NH_2_-functionalized polymer film and easily washed away by rinsing. Figure 3c, d shows AFM images of AuNWs aligned along the well-stretched DNAs on the NH_2_-functionalized polymer film. The average height of the DNA-templated AuNWs was 5.3 ± 0.5 nm. This height was similar to the average diameter of aniline-capped AuNPs, which were observed with the TEM, used in this experiment.

Figure 2b shows FT-IR spectra of the N_2 _plasma-treated polymer films that were synthesized by varying the RF power (20 to approximately 50 W) using the PECVD method and were treated in N_2 _plasma using the DBD method at a RF power of 150 W for 60 s. The spectra exhibit that the absorption peaks of N-H (at 3,400 cm^-1 ^and 1,650 cm^-1^) depended on plasma power, which was a parameter for polymer film synthesis, and those absorption peaks increased with an increase in the plasma power. It was reported that using high plasma power in polymer film synthesis has caused higher degrees of C = C bond contents due to high cross-linking between radicals of monomer molecule compared to using low plasma power [22]. Hence, it was expected that C = C bond, which has a π-bonding, can easily be broken by N_2 _plasma. This process can generate the cation radical at the surface and then more amine groups on surface of the plasma-polymerized film during plasma treatment using DBD process in N_2 _environment than the low plasma power.

Several types of experiments were carried out to prove that plasma power affects the alignment and immobilization of the DNA and the surface energy for the NH_2_-functionalized polymer films. First, the average coverage of the aligned DNA on the surface depends on the RF power. In a low power range (≤20 W), the negatively charged DNA molecules were weakly bound to the NH_2_-functionalized polymer thin film, and they were easily swept off from the surface during the alignment process (Figure 3e). However, a high power (≥40 W) caused the DNA molecules to be formed in bundles and random networks, and thus, they were not well stretched (Figure 3f). It was reported that the surface tension and the density of amine groups on the surface determined the degree of straightness and density of the aligned DNA molecules [10,12,23]. Therefore, it was considered that the proper surface treatment of the polymer film determined this aspect, and thus, the appropriate interaction between the surface of the NH_2_-functionalized polymer thin film and the DNA molecules would give excellent alignment of DNA molecules.

**Figure 4:**
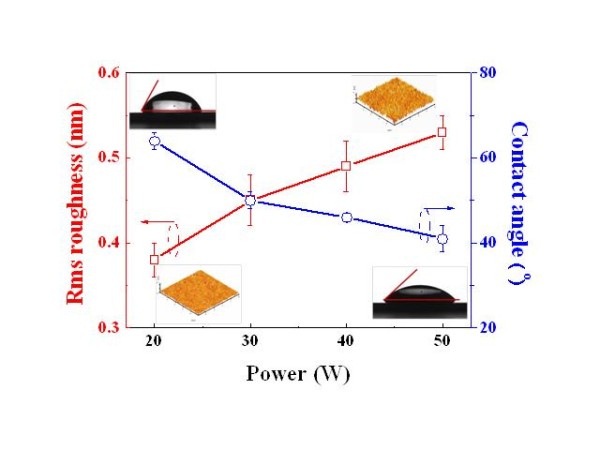
**Variation of rms roughness and contact angle for NH_2_-functionalized polymer film with plasma RF power**.

In addition, the surface energy was able to be controlled by RF power. The contact angle is one of the surface's sensitive properties that detects any changes at the modified surface. It is well known that the water contact angle increases as the surface energy is reduced. From the contact angle and AFM results, it was observed that the contact angle for the treated films decreased from 64° to 41°, but the root mean square [rms] roughness increased from 0.38 nm to 0.54 nm as the deposition power increased (Figure 4). This indicates that the N_2 _plasma-treated polymer films had higher surface energy with an increase in the deposition power. It was also demonstrated that such treatment and high plasma power were able to improve the ability of polymer films to be bound with other materials. Our results prove that RF power affects the density of the amine groups that are formed on the polymer thin films, and they play an important role in aligning and immobilizing the DNA on the NH_2_-functionalized polymer films.

## Conclusions

For the first time, we have proposed to use the NH_2_-functionalized polymer film for the alignment and the immobilization of DNA molecules for the purpose of applying them to nanodevices. The polymer film was deposited on a Si substrate by the PECVD method using a single molecular precursor, and the film was then treated using the DBD plasma surface treatment (or modification) method in N_2 _plasma under atmospheric pressure to form the amine groups on the surface. XPS and FT-IR spectra showed that the technique has been successful in forming nitrogen-containing groups by the N_2 _plasma, and this wasn't seen in the untreated material. The treated polymer surface was smooth enough (roughness was 0.45 ± 0.17 nm) to allow proper AFM measurements of the well-aligned DNA on the sample wafer. The DNA was well aligned and immobilized on the surface of the NH_2_-functionalized polymer films according to the tilting method. It was demonstrated that the N_2 _plasma treatment has been successful in forming the NH_2_-functionalized polymer films. In addition, the plasma power that was used to synthesize the polymer film played an important role in immobilizing the DNA on the NH_2_-functionalized polymer films as intended.

Therefore, we believe that the NH_2_-functionalized polymer film could be a promising material for organizing the DNA-based building blocks into structures that are needed for wiring and interconnecting functional nanodevices and/or biosensors. In addition, the fabrication technology could be easily adapted for arraying nanowires into more complex crossed structures and for making nanowires of other materials ordered.

## Competing interests

The authors declare that they have no competing interests.

## Authors' contributions

HJK and BH conceived the study. ISB, SJC, and JHB carried out the experiments. JH and IC contributed to the analysis study. BCL drafted the manuscript. All authors are involved in revising the manuscript and approved the final version.

## References

[B1] Nakao H Hayashi H Iwata F Karasawa H Hirano K Sugiyama S Ohtani T Fabricating and aligning π-conjugated polymer-functionalized DNA nanowires: atomic force microscopic and scanning near-field optical microscopic studies Langmuir 2005 21 7945 10.1021/la050145p 16089403

[B2] Braun E Eichen Y Sivan U Ben-Uoseph G DNA-templated assembly and electrode attachment of a conducting silver wire Nature 1998 391 775 10.1038/35826 9486645

[B3] Richter J Mertig M Pompe W Monch I Schackert HK Construction of highly conductive nanowires on a DNA template Appl Phys Lett 2001 78 536 10.1063/1.1338967

[B4] Harnack O Ford WE Yasuda A Wessels JM Tris(hydroxymethyl)phosphine-capped gold particles templated by DNA as nanowire precursors Nano Lett 2002 2 919 10.1021/nl020259a

[B5] Keren K Krueger M Gilad R Ben-Yoseph G Sivan U Braun E Sequence-specific molecular lithography on single DNA molecules Science 2002 297 72 10.1126/science.1071247 12098693

[B6] Porath D Bezryadin A de Vries S Dekker C Direct measurement of electrical transport through DNA molecules Nature 2000 403 635 10.1038/35001029 10688194

[B7] Nickels P Dittmer WU Beyer S Kotthaus JP Simmel FC Polyaniline nanowire synthesis template by DNA Nanotechnology 2004 15 1524 10.1088/0957-4484/15/11/026

[B8] Nakao H Hayashi H Yoshino T Sugiyama S Otobe K Ohtani T Development of novel polymer-coated substrates for straightening and fixing DNA Nano Lett 2002 2 475 10.1021/nl025528b

[B9] Washizu M Kurosawa O Electrostatic manipulation of DNA in microfabricated structures IEEE Trans Ind Appl 1990 26 1165 10.1109/28.62403

[B10] Zhang J Ma Y Stachura S He H Assembly of highly aligned DNA strands onto Si chips Langmuir 2005 21 4180 10.1021/la050129s 15835992

[B11] Nakao H Gad M Sugiyama S Otobe K Ohtani T Transfer-printing of highly aligned DNA nanowires J Am Chem Soc 2003 125 7162 10.1021/ja034185w 12797774

[B12] Deng Z Mao C DNA-templated fabrication of 1D parallel and 2D crossed metallic nanowire arrays Nano Lett 2003 3 1545 10.1021/nl034720q

[B13] Michalet X Ekong R Fougerousse F Rousseaux S Schurra C Hornigold N Slegtenhorst M Wolfe J Povey S Beckmann JS Bensimon A Dynamic molecular combing: stretching the whole human genome for high-resolution studies Science 1997 277 1518 10.1126/science.277.5331.1518 9278517

[B14] Shin M Kwon C Kim SK Kim HJ Roh Y Hong B Park JB Lee H Formation of *λ*-DNA's in parallel- and crossed-line arrays by molecular combing and scanning-probe lithography Nano Lett 2006 6 1334 10.1021/nl060160u 16834406

[B15] Kim HJ Roh H Hong B Controlled gold nanoparticle assembly on DNA molecule as template for nanowire formation J Vac Sci Technol A 2006 24 327

[B16] Heim T Mélin T Deresmes D Vuillaume D Localization and delocalization of charges injected in DNA Appl Phys Lett 2004 85 2637 10.1063/1.1794852

[B17] Nakao H Shiigi H Yamamoto Y Tokonami S Nagaoka T Sugiyama S Ohtani T Highly ordered assemblies of Au nanoparticles organized on DNA Nano Lett 2003 3 1391 10.1021/nl034620k

[B18] Wilsona DJ Rhodesb NP Williamsa RL Surface modification of a segmented polyetherurethane using a low-powered gas plasma and its influence on the activation of the coagulation system Biomaterials 2003 24 5069 10.1016/S0142-9612(03)00423-X 14568423

[B19] Kim HJ Roh Y Hong B Fabrication and characterization of DNA-templated conductive gold nanoparticle chains J Appl Phys 2009 105 074302 10.1063/1.3091281

[B20] Weibel DE Vilani b C Habert AC Achete CA Surface modification of polyurethane membranes using RF-plasma treatment with polymerizable and non-polymerizable gases Surf Coat Tech 2006 201 4190 10.1016/j.surfcoat.2006.08.050

[B21] Yang D-Q Poulin S Martinu L Microscale chemical and electrostatic surface patterning of Dow Cyclotene by N_2 _plasma Appl Surf Sci 2005 242 419 10.1016/j.apsusc.2004.09.077

[B22] Bae I-S Cho S-H Lee S-B Kim Y Boo J-H Growth of plasma-polymerized thin films by PECVD method and study on their surface and optical characteristics Surf Coat Technol 2005 193 142 10.1016/j.surfcoat.2004.07.022

[B23] Abramchuk SS Khokhlov AR Iwataki T Oana H Yoshikawa K Direct observation of DNA molecules in a convection flow of a drying droplet Europhysics Lett 2001 55 294 10.1209/epl/i2001-00412-2

